# Clinical Reflections

**Published:** 1883-03

**Authors:** Ad. Lippe

**Affiliations:** Philadelphia


### CLINICAL REFLECTIONS.

Ad. Lippe, M. D., Philadelphia.

On the evening of the 16th of December we were called to see a robust, well-developed girl six years old; had never before been very sick. Complains of sore throat and pain in the forehead, face flushed, pulse 120. On examining her throat I found great inflammation of the tonsils especially. She had complained of sore throat in the morning, and a lady physician at the time visiting her mother gave her some medicine in water, which the child declined to take, as it tasted very acid. It proved to be Carbolic acid, which had been administered to avoid a possible development of diphtheritis, then prevailing in some sections of the city. Gave one dose of Belladonna $^{cm}$ (Fincke). On the next morning I found her without fever and no headache, but right tonsil was much more inflamed ; she had slept some but had no appetite. Gave no medicine.

On the 18th I found her decidedly worse; swallowing much more painful, especially after a short sleep ; voice had the peculiar sound as in quinsy, the jaws were opened with difficulty, sides of the neck, especially the left, were very tender to the touch ; the neck was very stiff and painful. It was furthermore ascertained that her sore throat had begun on the left side, and that tonsil was now much more inflamed than before. Pulse 108. Refuses all food and takes only an occasional drink of milk. Gave one dose of Lachesis $^{5m}$ (Fincke), and ordered another dose to be dissolved in half a tumblerful of water at night if she should be very sleepless, which was done ; she took three doses, every hour one teaspoonful, and then obtained some sleep.

On the morning of the 19th she swallowed with more difficulty ; she was compelled to hawk a great deal, but detached only small quantities of tenacious mucus ; the neck was less stiff and not as painful. The Lachesis was only to be repeated at night if she were sleepless—but sleeping better she had no medicine.

On the 19th I examined her throat, and while depressing the root of the tongue the abscess broke and discharged very freely. No medicine.

On the 20th on examining the throat the diphtheritic condition had very rapidly developed itself, both tonsils and the uvula were covered by it, and complete loss of voice left no doubt that bacteria had already descended to the larynx ; there was no unpleasant odor from her throat and she was able to sit up, not complaining of weakness ; the hawking of mucus continued and she raised a ropy, stringy, very tenacious mucus ; pulse 108. The neck less stiff and painful. *One dose* of Kali bich. $^{cm}$ (Fincke) was now given.

On the 1st she felt better, no voice, but had raised great quantities of now perfectly loosened deposits ; some of them had bloody strings attached to them. No medicine.

On the 22d the hawking up had continued and she complained of great soreness of the throat when attempting to drink ; slept much better ; pulse 96. No medicine.

On the 23d her voice returned, hawked less, throat looked well but was very sore when she swallowed; the improvement continued from day to day. On the 26th she began to eat, and on the 30th of December I paid her my last visit, found her full of play, and she only asked for more food, and was therefore then consigned to the cook for further treatment. This day, the 1st day of February, she continues in perfect health, has regained all the flesh she lost during the prolonged abstinence from food, goes to school and enjoys it.

COMMENTS.—*First*, as to the treatment. There could have been no doubt as to the choice of the remedies at any time. The characteristic symptoms for the use of them are so clearly stated in that most excellent little work on diphtheria by Dr. Gregg that no one could have done anything else than was done in this case. There were no local applications made, no gargles used, as they have always proved to be pernicious in such grave cases of a disease, and are, furthermore, in antagonism to the strict tenets of our school. The dose was not repeated as long as the improvement continued, and in this case the last single dose administered did not exhaust its action till the last vestige of the disorder had disappeared; a violation of this rule—never repeat a medicine till the effect of the last dose administered has been fully exhausted—is invariably followed by bad consequences. As to the dose: at the last meeting of the American Institute the then President of the Institute recommended that "a limit" should be set as to the dose, and *HE* suggested to have the limit set at the 10th potency.

The leading advocates of restriction to liberty, apparently expressing opinions such as are in vogue among the Communists, address the profession through the columns of the New York *Medical Times*. There comes the veteran opponent to Hahnemann and his tenets and in an illogical manner proposes the separation of Dynamic and of Hahnemannism from sound Homœopathy. This veteran wants to have a law passed declaring that beyond the 12th potency there is no curative virtue; he boldly abuses all men who cure the sick by strict homœopathic treatment, of which art he has no conception. To his aid comes another man, by his own showing utterly ignorant of Homœopathy, its founder, and its history. This pair should be thanked, and are now publicly thanked, for the honest "warning" they gave such members of the Institute as have been persistent adherents to Hahnemann's teachings and have followed him faithfully, that they, on account of their fidelity to principles, must expect to be expelled by these base and ignorant pretenders from an Institute they created. Thanks for the timely warning. Whether the veteran pretender reads our reported cases or not, whether he denounces such reported cures as having been accomplished by any other means than reported, by some unaccountable manner, matters very little. Here are naked facts, and if the pretenders in Albany and Terre Haute can show better results by their eclectic practice we shall hereafter desist from publishing "homœopathic cures." Under the plea of unobstructed liberty of medical action, we claim the liberty of following Hahnemann, his tenets, and remain a homœopathic healer; and also the liberty to help to develop our healing art, to cure the sick and report such cures.

Two more cases of a similar character as the one above related have since been cured in a similar manner. In all former epidemics of diphtheria it was claimed that the characteristic symptoms of that form of disease were—great debility, formation of bacteria, offensive breath, and if loss of voice came it indicated the spreading of the diphtheritic deposits into the larynx, and that hardly three per cent. of the patients so attacked recovered. In this late epidemic we found first plain tonsilitis, followed by quinsy and then rapidly developed diphtheria; mortality, so far, none. As the characteristic symptoms of the former epidemic were absent, the great debility and the offensive breath being absent, but the quinsy sore throat preceding the development of the diphtheritic deposits being present in every case, it seems as if we should have a new pathology, just as we have new fashion plates, alternately issued at Berlin and Paris, to guide the medical men, just as the fashionable ladies are guided by the fashion plates. As homœopathic healers, we are not affected by these ever changing symptoms of even epidemically appearing diseases. Our law of cure, our strict adherence to principles and to the rules regulating the application of the law, will forever be to us an unerring guide in curing the sick.

In every one of these three grave cases so-called homœopathic remedies improperly chosen had been improperly administered. In a very large number of cases of sore throats here prevailing, one, and never more than two doses of medicines were all-sufficient to cure the sick.

The first section of Hahnemann's *Organon* reads: "*The first and sole duty of the physician is, to restore health to the sick. This is the true art of healing,*" and in this *Organon* of the healing art its founder gives his advice "how to do it." If the newly fledged philosophers at Albany and Terre Haute and their followers can do it better, why is it that after so much begging none of them ever published *one single case* in which they had followed Hahnemann's injunctions, and failing to cure resorted to their eclectic, freedom-born system, and then cured? Why is it so? Because they *never* knew what Homœopathy *is*. If they did, they would *cure* and be true healers, would need nothing better than what the developments of the true healing art brings us, and not wickedly try to pervert the true healing art into vile eclecticism.

